# *Drosophila* class-I myosins that can impact left-right asymmetry have distinct ATPase kinetics

**DOI:** 10.1016/j.jbc.2023.104961

**Published:** 2023-06-26

**Authors:** Faviolla A. Báez-Cruz, E. Michael Ostap

**Affiliations:** 1 Department of Physiology, and Center for Engineering Mechanobiology, Pennsylvania Muscle Institute, University of Pennsylvania Perelman School of Medicine, Philadelphia, Pennsylvania, USA; 2 Biochemistry and Molecular Biophysics Graduate Group, University of Pennsylvania Perelman School of Medicine, Philadelphia, Pennsylvania, USA

**Keywords:** myosin, kinetics, actin, ATPase, left-right asymmetry, chirality, vesicle transport

## Abstract

Myosin-1D (myo1D) is important for *Drosophila* left-right asymmetry, and its effects are modulated by myosin-1C (myo1C). *De novo* expression of these myosins in nonchiral *Drosophila* tissues promotes cell and tissue chirality, with handedness depending on the paralog expressed. Remarkably, the identity of the motor domain determines the direction of organ chirality, rather than the regulatory or tail domains. Myo1D, but not myo1C, propels actin filaments in leftward circles in *in vitro* experiments, but it is not known if this property contributes to establishing cell and organ chirality. To further explore if there are differences in the mechanochemistry of these motors, we determined the ATPase mechanisms of myo1C and myo1D. We found that myo1D has a 12.5-fold higher actin-activated steady-state ATPase rate, and transient kinetic experiments revealed myo1D has an 8-fold higher MgADP release rate compared to myo1C. Actin-activated phosphate release is rate limiting for myo1C, whereas MgADP release is the rate-limiting step for myo1D. Notably, both myosins have among the tightest MgADP affinities measured for any myosin. Consistent with ATPase kinetics, myo1D propels actin filaments at higher speeds compared to myo1C in *in vitro* gliding assays. Finally, we tested the ability of both paralogs to transport 50 nm unilamellar vesicles along immobilized actin filaments and found robust transport by myo1D and actin binding but no transport by myo1C. Our findings support a model where myo1C is a slow transporter with long-lived actin attachments, whereas myo1D has kinetic properties associated with a transport motor.

Myosin-Is are single-headed cytoskeletal motors that interact with actin filaments to carry out cellular functions related to membrane trafficking, dynamics, and organization (1). Recent studies have shown that two *Drosophila* myosin-I paralogs, myosin-1C (myo1C; also known as myo61F) and myosin-1D (myo1D; also known as myo31DF), are expressed in tissues that undergo left-right (L/R) asymmetry during development. L/R asymmetry is the process in early embryonic development that breaks the normal symmetry in the bilateral embryo and in flies occurs when organs such as the hindgut turn to become chiral (2, 3, 4, 5). Knockdown of myo1D results in *situs inversus* in chiral cells and tissues, and myo1C appears to negatively regulate myo1D activity when overexpressed (6, 7). Interestingly, overexpression of either paralog in nonchiral tissues results in cell and tissue chirality, with the handedness dependent on the paralog. Thus, myosin-Is are sufficient to induce cell, tissue, and organismal chirality in *Drosophila* (6, 8).

Directionality of induced L/R asymmetry in *Drosophila* tissues is determined by the identity of the myosin-I motor domain (6). Expression of protein constructs that contain the myo1D motor domain in the nonchiral epidermis or trachea results in dextral rotation of the tissue, and expression of the myo1C motor domain results in sinistral rotation (6). Interestingly, purified, recombinant myo1D turns actin filaments in a counterclockwise direction in *in vitro* actin gliding assays, whereas myo1C-powered actin filaments do not have a directional preference. While chiral turning of actin filaments by myosin may play a role in development of L/R asymmetry in cells and tissues, the lack of chirality from myo1C implies that it is not sufficient on its own, and so differences in the kinetics of the paralogs may also be important for differences in chiral development (9, 10).

In this study, we used transient and steady-state biochemical techniques to determine the rate constants that define the ATPase pathways of myo1C and myo1D (Fig. 1). Our goal was to test if there are differences in the ATPase pathways that could lead to functional specializations of the paralogs. We found the steady-state ATPase activities differ >10-fold. Importantly, we found that both motors have duty ratios >0.4 with the attachment lifetimes in force-bearing states differing by ∼9-fold. Even though both paralogs have high duty ratios, only myo1D could support transport of 50 nm vesicles *in vitro*. We propose that these kinetic differences could be pivotal for their function and dynamics in L/R asymmetry during *Drosophila* development.

**Figure 1:**
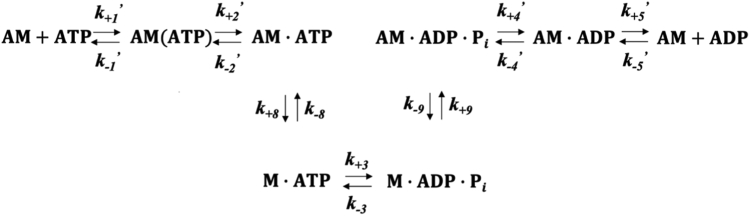
***Drosophila* myosin-I ATPase cycle**.

## Results

### Protein purifications of full-length myosin-Is and light-chain binding properties

**Figure 2:**
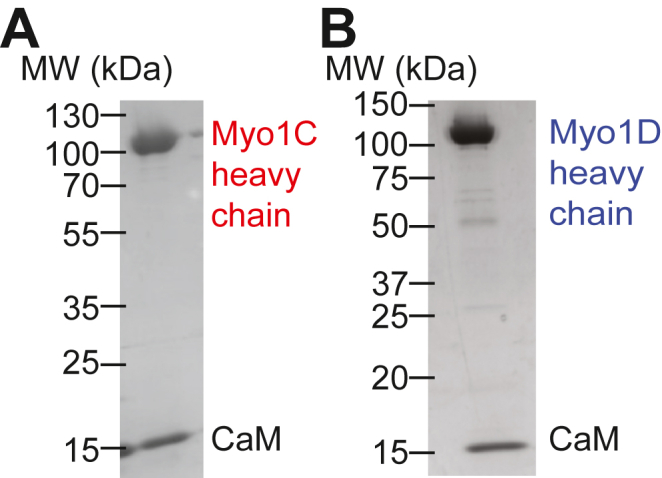
**Purified *Drosophila* myosin-Is and calmodulin light chain.***A* and *B*, a representative SDS-PAGE gel of (*A*) purified myo1C and (*B*) purified myo1D after anion exchange chromatography containing the heavy chain of 119 kDa and 117 kDa, respectively, and CaM light chain (16.7 kDa). myo1C, myosin-1C; myo1D, myosin-1D; CaM, calmodulin.

Recombinant full-length myo1C and myo1D constructs containing C-terminal Avi and FLAG tags were expressed using baculovirus in Sf9 insect cells and copurified with calmodulin (CaM) as described (6) (Fig. 2). Sequence analysis of myo1C and myo1D suggest the presence of three and two light-chain-binding IQ motifs, respectively (5). We determined the stoichiometry of the light chain to motor bound to each paralog by pulling down biotinylated myosin-Is with streptavidin beads and assessing CaM concentration by SDS-PAGE. We found an average of 2.8 ± 0.3 and 1.7 ± 0.2 light chains bind to myo1C and myo1D, respectively (Fig. S1), consistent with the number of available light chain binding sites for both paralogs. Non-CaM light chains were not found to copurify with either myosin.

### Myosin-Is steady-state ATPase activity

**Figure 3:**
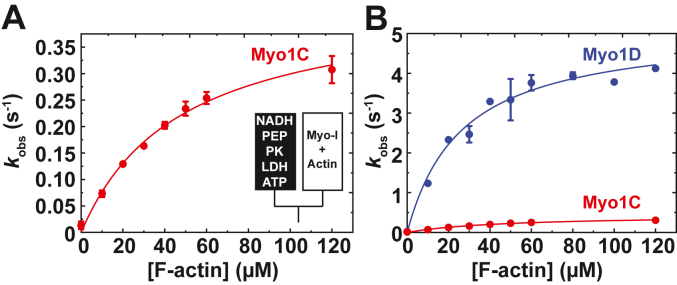
**Actin-activated steady-state ATPase activity.***A* and *B*, observed rate of 0.5 μM (*A* and *B*) myo1C or (*B*) myo1D (*blue*) with increasing F-actin concentrations. The *solid lines* represent the best fits by Equation 1 with a *V*_max_ of 0.44 ± 0.02 s^−1^ and 5.1 ± 0.3 s^−1^ and a *K*_ATPase_ of 48 ± 5 μM and 26 ± 4 μM, respectively. The error bars correspond to the standard deviation of three independent experiments using three different protein preparations per paralog. myo1C, myosin-1C; myo1D, myosin-1D.

The steady-state ATPase activities of myo1C and myo1D were determined using the NADH-coupled enzyme reaction in the presence of 0 to 120 μM phalloidin-stabilized F-actin. Actin substantially activates the ATPases activities of both paralogs (Fig. 3). The actin concentration dependence of the ATPase rates (*k*_obs_) was fitted by,

(1) $$k_{obs}=V_{o}+\left(\frac{V_{\max}\left[Actin\right]}{K_{ATPase}+\left[Actin\right]}\right)$$

where the actin concentration at half-maximum of the ATPase rate (*K*_ATPase_) rate is 48 ± 5 μM for myo1C and 26 ± 4 μM for myo1D, and the maximum ATPase rate (*V*_max_) is 0.44 ± 0.02 s^−1^ for myo1C and 5.1 ± 0.3 s^−1^ for myo1D (Table 1). Minimizing contaminating MgADP from protein preparations was crucial, as MgADP inhibited the ATPase rates due to its very tight actomyosin affinity (see below and Experimental procedures; (11)).

**Table 1:** Rate and equilibrium constants for key steps of *Drosophila* myo1C and myo1D ATPase cycle at 20º C^a^

Rate and equilibrium constants	Myo1C	Myo1D
*V*_o_^b^	0.01 ± 0.01 s^−1^	0.02 ± 0.02 s^−1^
*V*_max_^b^	0.44 ± 0.02 s^−1^	5.1 ± 0.3 s^-1^
*K*_ATPase_^b^	48 ± 5 μM	26 ± 4 μM
*k*_*+2*_′^c^	174 ± 5 s^−1^	179 ± 5 s^−1^
*K*_1_′^c^	73 ± 11 μM	118 ± 15 μM
*K*_1_′*k*_+2_′ (MgATP binding)^d^	2.4 ± 0.5 μM^−1^ s^−1^	1.6 ± 0.5 μM^−1^ s^−1^
*k*_+4_′ (P_i_ release)^e^	0.28 ± 0.03 s^−1^	27 ± 4 s^−1^
*K*_9_′ (M.ADP.P_i_ affinity)^d^	27 ± 7 μM	12 ± 5 μM
*k*_+5_′ (MgADP release)^c^	1.0 ± 0.1 s^−1^	8.7 ± 1.9 s^−1^
*K*_5_′ (MgADP affinity)^d^	89 ± 2 nM	44 ± 2 nM
*K*_i_^d^^f^ (MgADP IC_50_)	913 ± 90 μM	222 ± 88 μM

a KMg25 (10 mM Mops (pH 7.0), 25 mM KCl, 1 mM MgCl_2_, 1 mM EGTA, 1 mM DTT).

b NADH-coupled enzyme reaction.

c Pyrene-actin fluorescence.

d Calculated.

e MDCC-P_i_BP fluorescence.

f *In vitro* actin gliding assay.

### MgATP-induced pyrene-actomyosin-I dissociation

**Figure 4:**
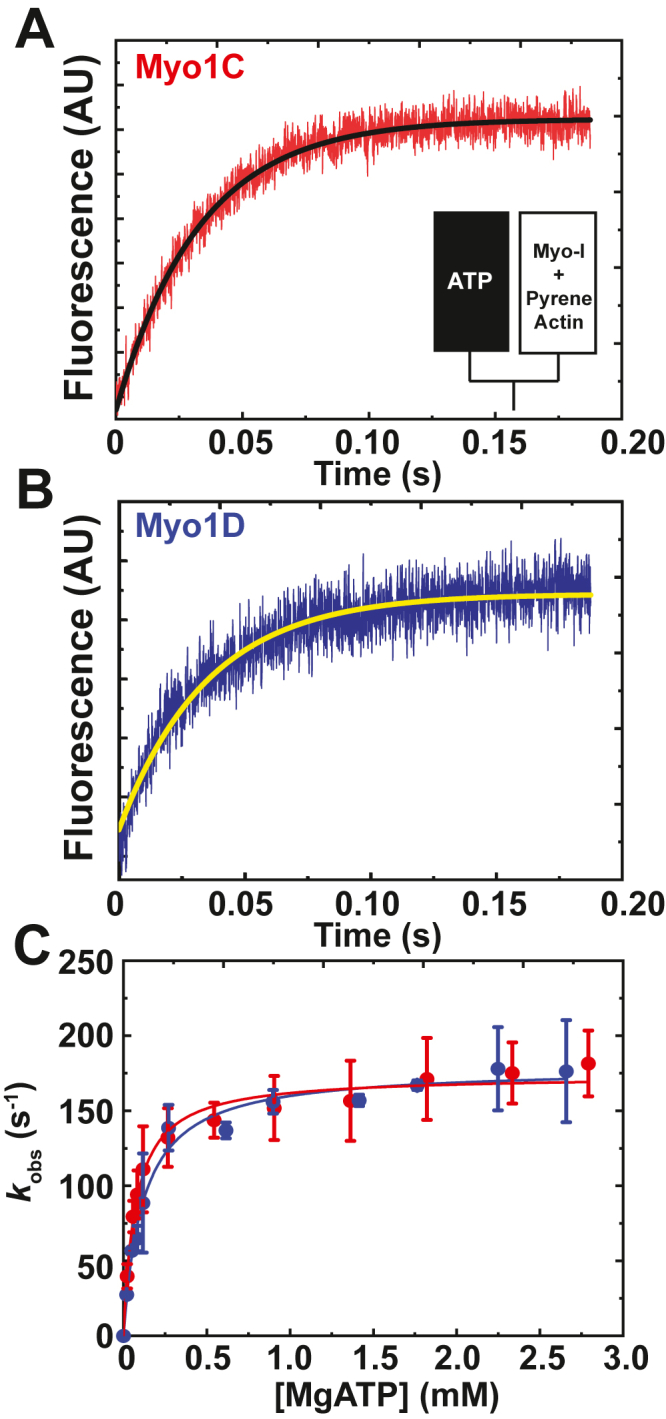
**MgATP-induced actomyosin-I dissociation.***A* and *B*, stopped-flow fluorescence transients of dissociation of (*A*) 0.5 μM myo1C and (*B*) 0.5 μM myo1D from 0.5 μM pyrene-actin-phalloidin by 20 μM MgATP. *The solid lines* represent single exponential fits to the fluorescence data with rates of (*A*) 30.3 ± 0.2 s^−1^ and (*B*) 28.6 ± 0.3 s^−1^. *C*, rate of MgATP-induced dissociation of myo1C (*red*) and myo1D (*blue*) from pyrene-actin-phalloidin as a function of MgATP concentration. The observed rates were obtained at each nucleotide concentration by fitting the stopped-flow data to a single exponential. The *solid lines* represent the best fits by Equation 2 with *K*_1_’= 73 ± 11 μM and 118 ± 15 μM, and *k*_+2_’= 174 ± 5 s^−1^ and 179 ± 5 s^−1^ for myo1C and myo1D, respectively. The error bars correspond to the standard deviation of three independent experiments using three different protein preparations per paralog. myo1C, myosin-1C; myo1D, myosin-1D.

**Figure 5:**
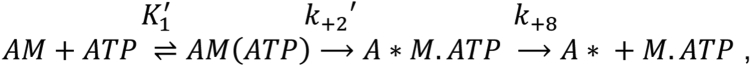
**Kinetic scheme for modeling MgATP binding**.

Pyrene-actin fluorescence was used to measure the MgATP-induced dissociation of actomyosin-I complexes and population of the weakly bound states (12). Mixing MgATP with myo1C or myo1D bound to pyrene-actin resulted in transient increases in fluorescence which were best fit by a single exponential function (Fig. 4, *A* and *B*). Rates (*k*_obs_) depended hyperbolically on the MgATP concentration, so the mechanism of MgATP-induced fluorescence enhancement was modeled as (Fig. 5; (12)). where *K*_1_′ is a rapid equilibrium binding step, *k*_+2_′ is a rate-limiting isomerization to the A∗M.ATP state, and *k*_+8_ is the rapid actin dissociation step. Data were fitted by:

(2) $$k_{obs}=\left[\frac{K_{1}^{\prime }\;\left[ATP\right]}{1+K_{1}^{\prime }\;\left[ATP\right]}\right]\;{k_{+2}}^{\prime }$$

The maximum rates of dissociation are similar for both myo1C (*k*_+2_′ = 174 ± 5 s^−1^) and myo1D (*k*_+2_′ = 179 ± 5 s^−1^), as were MgATP affinities, *K*_1_′ = 73 ± 11 μM for myo1C and *K*_1_′ = 118 ± 15 μM for myo1D (Fig. 4*C*). The apparent second-order rate constant for MgATP binding (*K*_1_′*k*_+2_′) for the two paralogs were calculated: 2.4 ± 0.5 μM^−1^s^−1^ and 1.6 ± 0.5 μM^−1^s^−1^ for myo1C and myo1D, respectively (Table 1).

### Actin-activated phosphate release

**Figure 6:**
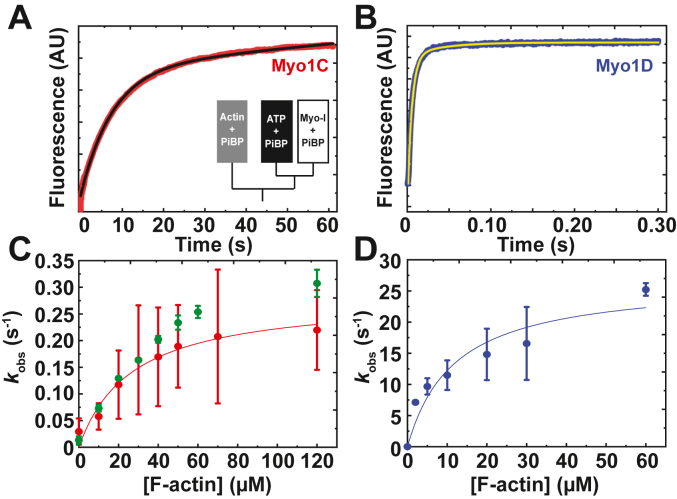
**Actin-activated phosphate (P**_**i**_**) release is the rate-limiting step for myo1C.***A* and *B*, stopped-flow fluorescence transients of Pi release from (*A*) 0.75 μM myo1C in the presence of 60 μM F-actin and 60 μM MgATP and (*B*) 0.5 μM myo1D in the presence of 60 μM F-actin and 50 μM MgATP. The observed rates were obtained at each F-actin concentration by fitting the stopped-flow data to a single exponential and slope for (*A*) and a single exponential for (*B*). *C* and *D*, rate of P_i_ release from (*C*) myo1C and (*D*) myo1D as a function of F-actin concentration. Values of the actin-activated steady-state ATPase of myo1C (*green points*) are added for reference in panel (*C*). The *solid lines* in (*C*) and (*D*) represent the best fits by Equation 3 with *k*_+4_’ = 0.31 ± 0.06 s^−1^ and 27 ± 4 s^−1^ and *K*_9_’ = 31 ± 14 μM and 15 ± 12 μM, respectively. The error bars correspond to the standard deviation of 3 to 8 independent experiments using three different protein preparations for myo1D and eight different protein preparations for myo1C. myo1C, myosin-1C; myo1D, myosin-1D.

**Figure 7:**
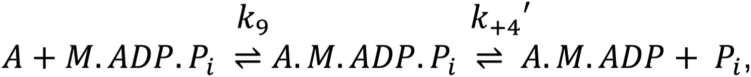
**Kinetic scheme for modeling Pi release**.

Phosphate binding protein (P_i_BP) fluorescently labeled with 7-diethylamino-3-((((2-Maleimidyl)ethyl)amino)carbonyl)coumarin was used to measure the actin-activated phosphate (P_i_) release rate (*k*_+4_′; Fig. 1) in sequential-mix, stopped-flow experiments (Fig. 6, *A* and *B*; 12). The rate of P_i_ release reports the transition of myosin from weak actin-binding to strong actin-binding states. Myo1C preincubated with apyrase-VII, to remove contaminating MgADP, was rapidly mixed with 60 μM MgATP and aged for 5 s to allow for MgATP binding and hydrolysis. The time course of phosphate release in the absence of actin followed a single exponential function with a rate of 0.03 ± 0.02 s^−1^. The observed rate increased hyperbolically with increasing actin, which was also pretreated with apyrase-VII (Fig. 6*A*). No lag was apparent in the transients, and a faster phase was not observed within the first second after mixing. The mechanism of the reaction was modeled as a two-step binding reaction as in Figure 7.

The maximum rate of P_i_ release obtained from fitting the observed rate *versus* actin by:

(3) $$k_{obs}=\left[\frac{K_{9}^{\prime }\left[Actin\right]}{1+K_{9}^{\prime }\left[Actin\right]}\right]\;k_{+4}^{\prime }$$

is *k*_+4_′ = 0.28 ± 0.03 s^−1^ for myo1C (Fig. 6*C*). The affinity of myo1C.ADP.P_i_ for actin filaments is *K*_9_′ = 27 ± 7 μM. Contaminating phosphate and competition for phosphate binding from the “phosphate mop” in the buffer during the very slow transient resulted in variability in the *k*_obs._ Nevertheless, we find the actin-dependent rate of P_i_ release rates to be similar to the actin-dependence of the steady-state ATPase activity, pointing to *k*_+4_′ as the rate-limiting step (Fig. 6*C*, green points).

Apyrase-treated myo1D was mixed with 60 μM ATP and aged for 5 s to allow MgATP hydrolysis. A range of 0 to 60 μM actin was then rapidly mixed with that MgATP-bound myosin. Phosphate release in the absence of actin had a rate of 0.02 ± 0.02 s^−1^, and *k*_obs_ increased hyperbolically with actin (Fig. 6*B*). The observed rapid phase of phosphate release was followed by a slow phase that is the result of multiple ATPase turnovers. The rates of the fast phase in the presence of actin were substantially faster than the myo1C transients. The rates of the fast phase were fit by Equation 3. The maximum rate of P_i_ release for myo1D is 96-fold faster than myo1C (*k*_+4_′ = 27 ± 4 s^−1^), and the affinity of myo1D.ADP.P_i_ for actin filaments is *K*_9_′ = 12 ± 5 μM (Fig. 6*D*). The maximum rate is more than 5-fold faster than *V*_max_, suggesting that the rate-limiting-step of myo1D occurs after P_i_ release.

### MgADP release

**Figure 8:**
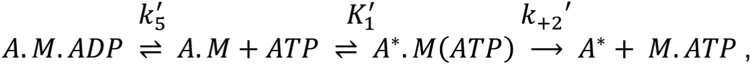
**Kinetic scheme for modeling MgADP release**.

The rate of MgADP release (*k*_+5_′) was determined by MgATP-induced dissociation of actomyo1C.ADP and actomyo1D.ADP from pyrene-actin as shown in Figure 8.

**Figure 9:**
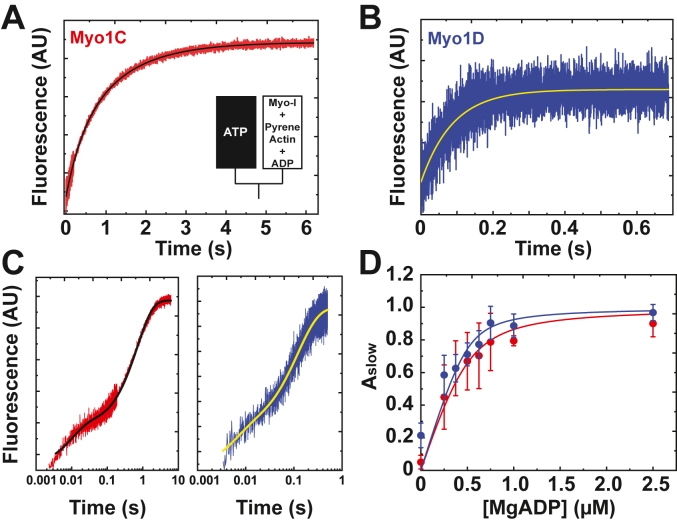
**Determination of the rate of MgADP release from myo1C and myo1D.***A* and *B*, stopped-flow fluorescence transients of MgATP (1 mM)-induced dissociation of (*A*) 0.5 μM pyrene-actomyo1C and (*B*) 0.5 μM pyrene-actomyo1D each preincubated with 2.5 μM MgADP. The solid lines represent single exponential fits to the fluorescence data with rates of (*A*) *k*_5+_’ = 1.0 ± 0.1 s^−1^ and (*B*) 8.7 ± 1.9 s^−1^. *C*, log scales of stopped-flow transients of 0.5 μM actomyo1C (*red*) and actomyo1D (*blue*) preincubated with 0.5 μM MgADP mixed with 1 mM MgATP. Transients are composed of a fast phase corresponding to MgATP-induced actomyosin-I dissociation, followed by a slow phase corresponding to MgADP release. *D*, MgADP affinity (*K*_5_’) of myo1C (*red*) and myo1D (*blue*) dependent on nucleotide concentration. The observed rate constants were obtained at each nucleotide concentration by fitting the stopped-flow data to a square fit equation. The *solid lines* represent the best fits by Equation 4 with *K*_5_’ = 89 ± 2 nM for myo1C and 44 ± 2 nM for myo1D (11). The error bars correspond to the standard deviation of three independent experiments using three different protein preparations per paralog. myo1C, myosin-1C; myo1D, myosin-1D.

When myosin active sites are saturated with MgADP, the rate of MgATP-induced dissociation of actomyosin is limited by the slow dissociation of MgADP (11). MgADP (2.5 μM; see below) was preincubated with 0.5 μM pyrene-actomyosin-I and mixed with 1 mM MgATP. Fluorescence transients were best fit by single exponential functions. The rate of MgADP release (*k*_+5_′ = 1.0 ± 0.1 s^−1^) from actomyo1C is ∼2-fold faster than *V*_max_, and rate of MgADP release from actomyo1D (*k*_+5_′ = 8.7 ± 1.9 s^−1^) is similar to *V*_max_, suggesting that this step is rate-limiting for myo1D (Fig. 9, *A* and *B*).

In the presence of subsaturating MgADP concentrations, MgATP-induced pyrene-actomyosin fluorescence transients were best modeled as the sum of two exponential components: a fast component resulting from MgATP binding to nucleotide-free actomyosin-I and a slow component that is the result MgADP dissociation (*k*_+5_′) (Fig. 9*C*). The affinity of MgADP for pyrene actomyosin (*K*_5_′) was determined by measuring the fraction of the amplitude corresponding to the slow component (A_slow_) as a function of MgADP concentration (Fig. 9*D*). The high affinity of MgADP for myosin required fitting by the quadratic equation,

(4) $$y=\frac{\left(\left[ADP\right]+\left[Myosin\right]+K_{5}^{\prime }\right)-\sqrt{{\left(\left[ADP\right]+\left[Myosin\right]+K_{5}^{\prime }\right)}^{2}-4\;\left(\left[ADP\right]*\left[Myosin\right]\right)}}{\left(2*\left[Myosin\right]\right)}$$

where *y* is the fraction of myosin-I bound to MgADP (12). These myosin-Is have nanomolar affinity for MgADP (*K*_5_′): 89 nM for myo1C and 44 nM for myo1D, which are among the tightest MgADP affinities observed for characterized myosins (13, 14, 15, 16).

### Duty ratio

The duty ratio is defined as the fraction of the ATPase cycle in which myosin dwells in actin-attached, force bearing states. Our kinetic characterization points to MgADP release as the step that limits exit from the strongly bound states; thus, we can estimate the duty ratio as:

(5) $$Duty\;Ratio=\frac{Lifetime\;of\;AM.ADP\;state\;\left(\frac{1}{k_{+5}^{\prime }}\right)}{ATPase\;cycle\;time\;\left(\frac{1}{V\;\max}\right)}$$

The calculated myo1C (0.44) and myo1D (0.59) duty ratios at saturating actin concentrations are substantially higher than other characterized myosin-I paralogs (1, 10, 14, 15, 17).

### Actin gliding assays

**Figure 10:**
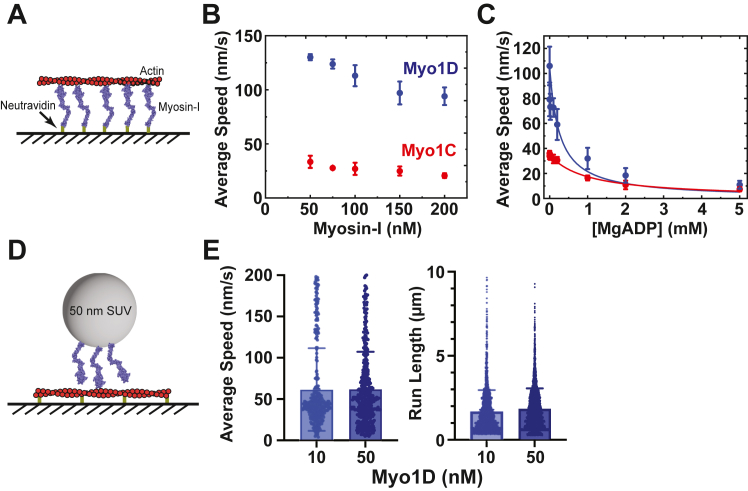
**Actin gliding and SUV motility assays.***A*, schematic of experimental settings for actin gliding assays with surface-immobilized myosin-I attached *via* biotin-neutravidin. *B*, biotinylated myosin-Is show average velocities decrease as a function of higher motor concentration. *C*, MgADP concentration-dependence of actin motility in the presence of 2 mM MgATP. The error bars for *B* and *C* correspond to the standard deviation of 3 to 6 independent experiments with one different protein preparation per paralog. *D*, schematic of SUV motility assay on immobilized actin filaments. Myo1D is attached to the SUV *via* the PtIns (4, 5)P_2_ – tail interaction. *E*, average speeds and run lengths for three independent experiments of (*light blue*) 10 nM or (*dark blue*) 50 nM myo1D bound to SUVs. myo1C, myosin-1C; myo1D, myosin-1D; SUVs, small unilamellar vesicles.

To assess motor activity, we carried out actin gliding assays by attaching a range of biotinylated myosin-I concentrations (50 nM – 200 nM) to neutravidin adsorbed to a glass coverslip (Fig. 10*A*; see Experimental procedures). Myo1C propelled actin filaments with an average velocity of 33 ± 6 nm/s, while myo1D propelled filaments with an average velocity of 130 ± 3 nm/s, consistent with kinetics showing myo1D is a faster motor than myo1C. Increasing the motor concentration in the motility chamber resulted in decreases in the average velocities of myo1C (21 ± 2) and myo1D (94 ± 8 nm/s; Fig. 10*B*). The increase of motor concentration likely results from multiple motors attaching to a single filament at higher densities, inhibiting actin gliding. Both myosin-Is slow with increasing motor density (Fig. S2), suggesting mechanical load slows gliding; however, further mechanochemical studies need to be performed to assess force sensitivity (18, 19, 20, 21, 22, 23). The observed gliding velocities in this experimental setting are faster compared to velocities on fluid lipid bilayer substrates (6), and circular actin gliding promoted by myo1D is attenuated due to rigid attachment to nitrocellulose, as previously described (23).

Kinetically, we observe that both myosin-Is have nanomolar affinity for MgADP (Fig. 9; Table 1), so we explored the effect of MgADP on actin gliding speeds. We observed a sharp MgADP concentration-dependent decrease in gliding velocity in the presence of 2 mM MgATP (Fig. 10*C*). The data were fitted by the following equation to determine the MgADP inhibition constant (*K*_i_, (24)):

(6) $$v_{obs}=\frac{v_{0}}{1+\left(\frac{\left[ADP\right]}{K_{i}}\right)}$$

where *v*_0_ is the gliding velocity in the absence of MgADP and was fixed at 36 nm/s for myo1C 110 nm/s for myo1D. Fits yielded *K*_i_ values of 913 ± 90 μM and 222 ± 88 μM for myo1C and myo1D, respectively (Table 1), which indicates that MgADP effectively competes with 2 mM MgATP for binding.

### *Drosophila* myo1D supports small unilamellar vesicles trafficking

Our kinetic characterization indicates that myo1C and myo1D have high duty ratios, which may suggest an ability to transport cargo at low densities. Thus, we tested the ability of myo1C and myo1D to transport small unilamellar vesicles (SUVs) created by extrusion through 50 nm filters composed of 4% PtdIn (4,5)P_2_, 96% 18:1 (Δ9-*Cis*) PC, and 0.04% lissamine rhodamine-labeled PE along immobilized actin filaments (Fig. 10*D*; see Experimental procedures). Mixing 10 nM or 50 nM myo1D with 5 μM SUVs resulted in processive vesicle motility, with many vesicles traversing the entire length of actin filaments and switching tracks (Movie S1). Automated fluorescent particle tracking revealed experiments at both concentrations had similar average speeds and run lengths of the vesicles at (53 ± 32) nm/s and (1.8 ± 1.3) μm (Fig. 10*E*). Addition of 10 nM myo1C showed no vesicle motility, although occasional actin binding was observed (Movie S1) and increasing motor concentration to 50 nM increased actin binding but did not support motility. These data suggest that myo1D is a *bona fide* high duty ratio motor that can support vesicle trafficking even at low motor concertation, while myo1C can support limited vesicle binding to actin but not promote motility under the same concentrations.

## Discussion

### Myo1C and myo1D have substantially different biochemical kinetic properties

In this study, we define key kinetic rate constants and establish important biochemical differences between two myosin-I paralogs that have opposing roles in cell and tissue chirality in *Drosophila*. Myo1C and myo1D share the same ATPase pathway found for other myosins (9, 10, 12, 25), but the kinetic rate constants that define their pathways result in the two motors having distinct motile properties (Table 1). Kinetic differences include: (1) >10-fold faster *V*_max_ for myo1D, (2) phosphate release as the rate-limiting step from actomyo1C and MgADP release from actomyo1D, (3) ∼9-fold faster rate of MgADP release from actomyo1D, and (4) a greater duty ratio for myo1D.

The different duty ratios and attachment lifetimes of the two myosins likely reflect enzymatic adaptations that evolved for specific biological functions. Thus, a notable finding is that although myo1D has a higher duty ratio than myo1C, the actin-attachment lifetime of myo1C (∼1 s) is predicted to be ∼9-fold longer than myo1D (∼110 ms). Additionally, calculated actin-attachment rates of the M.ADP.P_i_ states (*k*_+4_′/*K*_9_′; Fig. 6) are substantially different for myo1C (0.010 μM^−1^s^−1^) and myo1D (2.3 μM^−1^s^−1^). These parameters suggest that myo1D may be suited for processes that include rapid actin attachment and detachment kinetics or vesicle transport, whereas myo1C may be suited for sustained tension maintenance. This prediction agrees with our finding that myo1D can processively transport 50 nm vesicles along single actin filaments, but myo1C cannot under experimental conditions (Movie S1). Additionally, myo1D motors will detach from actin filaments much more rapidly, possibly relieving tension in the actin cytoskeleton (see below).

Myo1D moves actin filaments in gliding assays at a greater rate than myo1C but not to the extent predicted by the ∼9-fold difference in the rate of MgADP release. This discrepancy in rates could be explained by differences in lever arm compliance. It is also possible that the speed of myo1C is limited by slow transition rate of the motor to the strong binding states (26). Further mechanochemical studies such as optical trapping experiments and structural studies such as cryo-EM are necessary to address this question.

### Relating myosin-I biochemistry to cellular function

During development in *Drosophila*, class-I myosins are transiently expressed in tissues that undergo L/R asymmetry, and knocking out myo1D results in *situs inversus* (2, 4, 6). The mechanisms by which myosin-I paralogs participate in cell and tissue chirality have not been determined. However, there are two important facets to the myosin-dependent chirality mechanisms. First, the presence of either myo1C or myo1D is sufficient to induce chirality in nonchiral tissues (6). Second, the properties of the motor domain determine the chiral direction, so there must be an intrinsic property of the motors that lead to chirality and the differences in turning direction. Although we showed previously that the motors have differences in the ability to power asymmetric gliding of actin filaments *in vitro* when bound to fluid bilayers (6), it is not known if this turning activity is important for cell chirality. In this study, we revealed that there are substantial differences in the ATPase kinetics of the two motors (Table 1). We propose that these differences in mechanochemistry are important for their differing abilities to promote L/R asymmetry.

Cell biological and biochemical experiments in *Drosophila* suggest a direct link between myosin-I and cell–cell adhesion proteins, DE-cadherin and β-catenin (7). In this role, myosin-Is may work as a tether in conjunction with other actin cytoskeleton components to induce chirality. Notably, the formin DAAM has been shown to be essential for L/R asymmetry development in *Drosophila*, where it interacts with both myo1C and myo1D (27). Thus, the interaction between myosin-I and the DAAM-dependent F-actin network appears to create the chiral cytoskeleton.

It has been proposed that vertebrate formin proteins induce cell chirality through their spinning at the actin filament end as a result of their processive elongation activity (28, 29). Torque generated by this spinning has been modeled to be transmitted to the cellular actin network to induce chirality *via* the cross-linking protein, alpha-actinin 1. Strikingly, varying the expression levels of alpha-actinin 1 changes the handedness of cell chirality (28, 30), which is likely the result of altered attachment lifetimes between the cytoskeletal linkages. These altered kinetics are proposed to result in changes in cellular twisting direction (30). Given the >8-fold differences in the actin-attachment lifetimes of myo1C and myo1D, as determined by MgADP release rates (*k*_+5_′), we propose that myosin-I may be playing a similar role in communicating the chiral activity of DAAM to the *Drosophila* cytoskeleton by crosslinking actin to adhesion proteins and/or the cell membrane.

Alternatively, myosin-I may have a role in cadherin transport and membrane recycling that affects generation of L/R asymmetry. In *Drosophila*, knockdown of myo1D expression leads to a decrease in Rab-11 endosomes and less E-cadherin in the cell–cell contacts in the hindgut (31). Given our finding that myo1D is capable of powering vesicle transport *in vitro*, it is possible that in conjunction with formin activity, changes in myo1D-dependent trafficking could transport and concentrate E-cadherin at the adhesion site which may affect chirality. Interestingly, although not a planar cell polarity mechanism, the vesicle transport activity of myo1D is believed to be important for the development of nodal flow structures and development of L/R asymmetry in zebrafish (32, 33). Further cell- and organism-based experiments designed to alter the myosin kinetics should help distinguish between these possibilities.

## Experimental procedures

### Proteins and solutions

Full-length *Drosophila* myo1C (Ostap Lab construct number pLT49) and myosin-1D (Ostap Lab construct number pLT47), with C-terminal FLAG-Avi tags, were expressed as previously described (6). Sf9 or HI5 cell cultures (1–2 L) infected with baculovirus were pelleted, washed, flash frozen in liquid nitrogen, and stored at −80 °C for subsequent purification. For purification, pellets were lysed using a cell homogenizer with 1X lysis buffer (0.5% IGEPAL, 200 mM NaCl, 10 mM Tris-HCl pH 7.5, 4 mM MgCl_2_, 1 mM EGTA, and 1 mM β-mercaptoethanol), 1 M DTT, 1 mM PMSF, 10 μg/ml leupeptin-aprotinin, and 2 mM ATP. Lysed cells were centrifuged at 162,005*g* (Ti70.1 rotor, Beckman Coulter) for 1 h at 4 °C. Supernatant was flowed through a 2 ml anti-FLAG (anti-DYKDDDDK G1 Affinity resin, GenScript) column for binding, extract from 1 L culture for 1 × 10 mm column. Columns were then washed with 1× wash buffer (0.2 M NaCl, 0.01 M Tris-HCl pH 7.5, 4 mM MgCl_2_, 1 mM EGTA, 1 mM β-mercaptoethanol), 1M DTT, 1 mM M PMSF, and 10 μg/ml leupeptin-aprotinin. Elution buffer (200 mM NaCl, 10 mM Tris-HCl pH 7.5, 1 mM EGTA, 1 mM DTT, 0.2 mg/ml FLAG peptide, 5 μM recombinant chicken CaM, 10 μg/ml leupeptin-aprotinin) was added to the columns and incubated for 90 min to elute myosin-I proteins. Fractions containing myosin-Is were bound to a Mono Q column, and the respective protein was eluted at approximately 580 to 680 mM KCl using a 0 to 70% gradient of a high salt buffer (10 mM Tris-HCl pH 7.5, 1 M KCl, 1 mM EGTA, 1 mM DTT). Once protein presence was confirmed through SDS-PAGE, the fractions were pooled and dialyzed overnight at 4 °C against KMg100 buffer (10 mM Mops pH 7.0, 100 mM KCl, 1 mM EGTA, 1 mM MgCl_2_, 1 mM DTT, and 50% glycerol). We found protein concentrations >4 mg/ml resulted in protein precipitation. Protein was stored in 50% glycerol at −20 °C to be used for kinetic experiments. Myosin-I concentration was determined through Bradford Colorimetric assay (Bio-Rad, #5000006) using BSA standards (Albumin Standard from ThermoScientific, #23209). Proteins used for *in vitro* gliding assays on lipid bilayer substrates were not dialyzed in the presence of glycerol. Rather, myosin-I was aliquoted after elution from Mono Q and stored in liquid nitrogen.

Myosin-I constructs were biotinylated at the C-termini Avi tag sequence using BirA biotin-protein ligase (Avidity). Supernatants collected post-FLAG columns were spin-concentrated using Amicon Ultra Centrifugal Filters (100 kDa cut-off) for 10 min until the protein volume was decreased 3-fold. BirA enzyme (10 μl of 3 mg/ml) and Biomix B buffer were added to final concentrations of 10 mM ATP, 10 mM Mg_2_^+^ acetate, 50 μM d-biotin (Avidity). The mixture was incubated at 25 °C for 30 min, followed by final purification on a Mono Q column. Myosin-I purity was confirmed through SDS-PAGE gel, and the protein was dialyzed against KM100 + 50% glycerol buffer as described above. Biotinylation was confirmed through chemiluminescence (SuperSignal West Pico PLUS Chemiluminescent Substrate; Thermo #34580) by HRP-streptavidin (Thermo, #21130).

Rabbit skeletal muscle actin was purified as previously described (34) and used without further modification for steady-state ATPase and phosphate release experiments. Pyrene-labeled F-actin was prepared as described (35, 36). Both unlabeled and pyrene-labeled actin filaments were dialyzed against KMg25 buffer (10 mM Mops pH 7.0, 25 mM KCl, 1 mM EGTA, 1 mM MgCl_2_, and 1 mM DTT) to minimize free nucleotide. To further decrease the nucleotide contamination, both unlabeled and labeled actin were treated with saturating phalloidin concentrations (Cayman Chemicals, #18039) to stabilize filaments, and the stabilized filaments were sedimented at 146,944*g* for 45 min at 4 °C. Pellets containing the filaments were resuspended in KMg25 and treated with Apyrase-VII (Sigma Aldrich, A6535) and incubated for 30 min at 25 °C to remove free nucleotides. The F-actin was then centrifuged as above, and the pellet was resuspended in KMg25 and dialyzed against the same buffer overnight. Dialyzed F-actin was collected and stored on ice until used for experiments. Nucleotide concentrations for stopped-flow experiments were determined spectrophotometrically (ε_259_ = 15,400 M^−1^cm^−1^).

### Stopped-flow kinetics

A stopped-flow apparatus (SX20 Stopped Flow Spectrometer) was used to acquire all transients. The dead time of the instrument is <3 ms with a 120-μl sample volume. Fluorescence excitation was provided by a 100-W Hg lamp. For steady-state ATPase activity, NADH absorbance was monitored at 340 nm. Pyrene-actin for MgATP-induced actomyosin-I dissociation and MgADP release was excited at 365 nm, and the fluorescence emission peak was detected using a 405 nm long-pass filter. For actin-activated phosphate release, fluorescently labeled mutant phosphate binding protein (MDCC-labeled PiBiP; (7-diethylamino-3-((((2-maleimidyl)ethyl)amino)carbonyl) coumarin)-labeled phosphate binding protein) was excited at 430 nm, and fluorescence was detected with a 440 nm long-pass filter (12, 37). Data were acquired and analyzed using Pro Data-SX software. Stopped-flow data were fitted to exponentials functions by a nonlinear least-squares curve fitting. All the reagent concentrations reported are postmixing. For Pi release experiments, 375 nM–3 μM myo1C and 375 to 500 nM of myo1D were used.

### Actin gliding assays

*In vitro* actin gliding assays were conducted in KMg25 buffer (also known as Motility Buffer 1X). Purified G-actin (34) was polymerized using the same buffer and stabilized with Rhodamine Phalloidin (Invitrogen, R415). Recombinant chicken CaM was expressed and purified as previously described (38).

The actin gliding assays on streptavidin-biotinylated myosin-I were performed at room temperature (20–22 °C) with 0.5% nitrocellulose-coated glass (collodion 2% dissolved in amyl acetate, electron microscopy, #12620-30; amyl acetate, Electron Microscopy Catalog, #10815). The order of reagents introduced to the chamber was as follows: 0.1 mg/ml neutravidin (ThermoFisher Scientific #31000), 2 mg/ml BSA (Sigma Aldrich A7906), 50, 75, 100, 150, or 200 nM biotinylated myo1C or myo1D, and two washes with 2 mg/ml BSA + 5 μM CaM, The final assay solution contained 5 nM Rhodamine phalloidin-stabilized actin filaments, 2 mM MgATP, 5 μM CaM, 5 mg/ml glucose, 92 U/ml glucose oxidase (Sigma), and 48 mg/ml catalase from bovine liver (Roche) and BSA to avoid nonspecific binding in KMg25. In experiments that required it, MgADP was added to the final assay solution.

The open ends of the motility chamber were sealed using vacuum grease to prevent drying while acquiring data. Fluorescent actin filaments were visualized using a Leica DMIRB microscope with a 100-fold magnification Leica oil-immersive objective of numerical aperture 1.4. The leading edge of actin filaments were tracked using the Manual Tracking plugin from ImageJ (39), and the average speeds were determined *via* displacement over time using Microsoft Excel.

### Small unilamellar vesicle motility assays

Fluorescent SUVs with ∼50 nm diameter were generated by mixing of 4% phosphatidylinositol 4,5-bisphosphate (PI(4,5)P_2_, Avanti Polar Lipids, #850155) and 0.04% 18:1 Liss Rhod PE (Avanti Polar Lipids, #810150), and 96% 18:1 (Δ9-*Cis*) PC (Avanti Polar Lipids, #850375) was used to create lipid bilayers as previously described (6, 23).The lipid mixture was extruded through a 50 nm filter using a lipid extruder (Avanti). SUVs were kept at room temperature under nitrogen and covered in aluminum foil to avoid light exposure until use.

An immobilized actin network in a flow chamber to assay SUV motility was created by flowing 0.2 mg/ml neutravidin, followed by two washes of 2 mg/ml BSA and followed by 100 nM F-actin stabilized with 90:10 Alexa-488:Biotin Phalloidin (Alexa-488 Phalloidin; ThermoFisher Scientific A12379 and Biotin Phalloidin; Thermo Fischer Scientific B7474) into a 0.5% nitrocellulose-coated coverslip.

Specified concentrations of myosin-Is were preincubated with 5 μM SUVs to ensure binding *via* myosin-I tail domain with PI(4,5)P_2_ (6, 23, 40). The mixture was added to a solution containing final concentrations of 5 μM CaM, 2 mM MgATP, 20 mg/ml glucose oxidase, 4 mg/ml catalase, 5 mg/ml glucose, and BSA to avoid nonspecific binding in KMg25. The final solution added to chambers containing the immobilized actin network, and the chamber was sealed with vacuum grease. Image stacks were acquired by fluorescence microscopy at a rate of 1 frame/second for 5 min. Image states were processed using the Cega filtering program (41) and the Trackmate plugin from ImageJ (39).

## Data availability

Data to be shared upon request to E. Michael Ostap (ostap@pennmedicine.upenn.edu).

## Supporting information

This article contains supporting information (5).

## Conflict of interest

The authors declare no conflict of interest with the contents of this article.
