# Supplementary material for: Drosophila class-I myosins that can impact left-right asymmetry have distinct ATPase kinetics
Source: J Biol Chem. 2023 Jun 26;299(8):104961. doi: 10.1016/j.jbc.2023.104961 (PMC10374968; doi:10.1016/j.jbc.2023.104961)
Supplement: Supporting Information [file mmc2.pdf]

SUPPORTING INFORMATION

Drosophila class-I myosins that can impact Left-Right asymmetry have distinct ATPase kinetics

Faviolla A. Báez-Cruz[1,2] & E. Michael Ostap[1,3]

1. Pennsylvania Muscle Institute, Department of Physiology, and Center for Engineering Mechanobiology, University of Pennsylvania Perelman School of Medicine, Philadelphia, PA, USA
2. Biochemistry and Molecular Biophysics Graduate Group, University of Pennsylvania Perelman School of Medicine, Philadelphia, PA, USA
3. Address correspondence to E.M. Ostap (ostap@pennmedicine.upenn.edu): Pennsylvania Muscle Institute, 700A Clinical Research Bldg., 415 Curie Blvd., Philadelphia, PA 19104.

<u>Two Supporting Figures:</u>

Figure S1. Myosin-I: CaM binding ratio.

Figure S2. Normalized Average Speeds for biotinylated myosin-Is actin gliding assays.

<u>One Supporting Movie:</u>

Movie S1: Video showing 10 nM myo1C and 5μM SUV (magenta; left) and 10 nM myo1D and 5 μM SUV (magenta; right) in the presence of immobilized actin filaments (green).

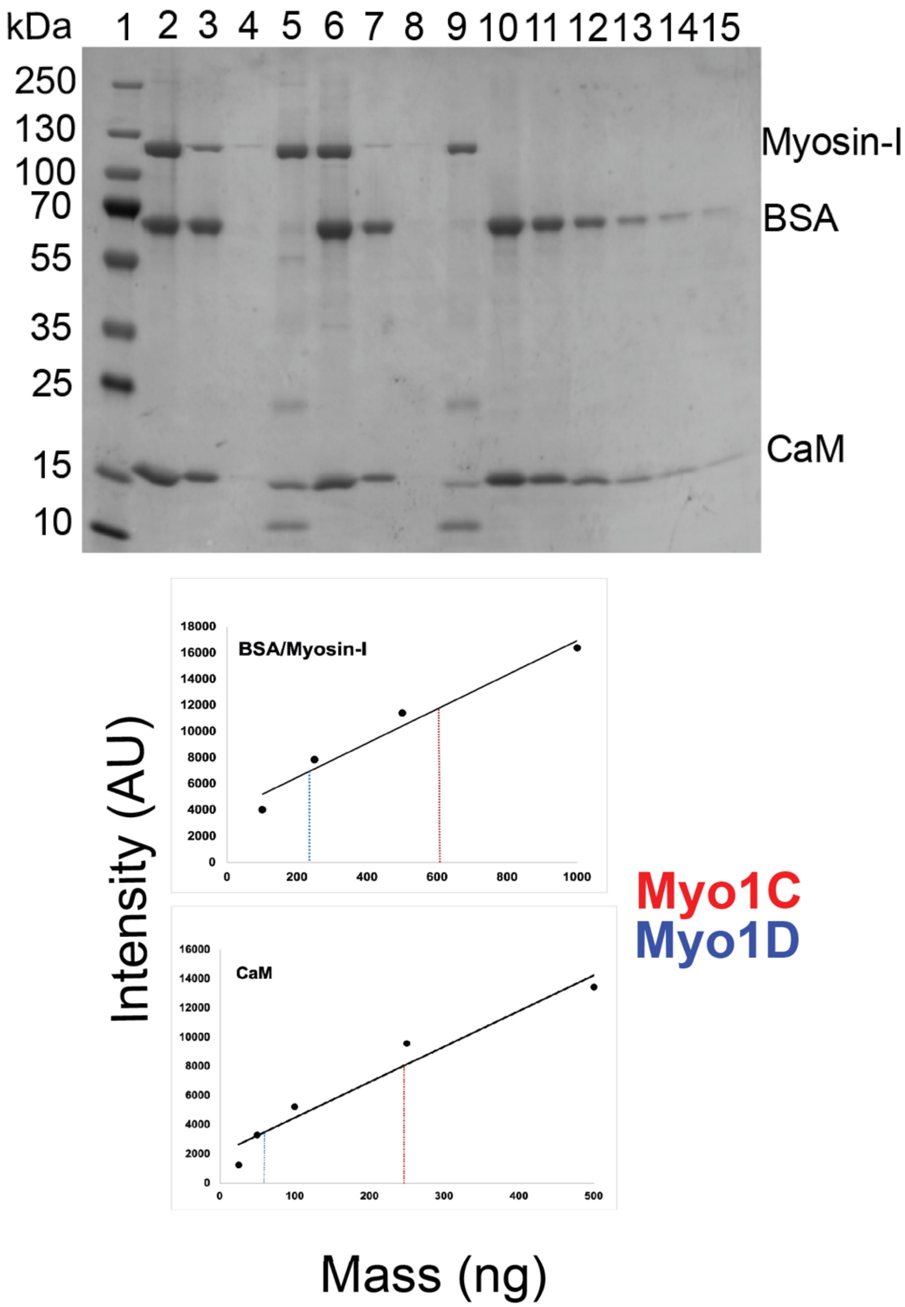

Figure S1. Myosin-I: CaM binding ratio. SDS-PAGE gel of representative experiment to measure the ratio of myosin-I to calmodulin. Biotinylated myosin-Is were incubated with excess CaM and streptavidin beads, washed, and beads were subjected to SDS-PAGE. Lanes on the gel: 1) MW: molecular weight, 2) Starting myo1C, 3) Supernatant myo1C, 4) Wash myo1C, 5) Final myo1C, 6) Starting myo1D, 7) Supernatant myo1D, 8) Wash

myo1D, 9) Final myo1D, 10-15) BSA and CaM standards. Results show 1 myo1C binds to 2.8 CaM, and 1 myo1D binds to 1.7 CaM as expected from previous data (1).

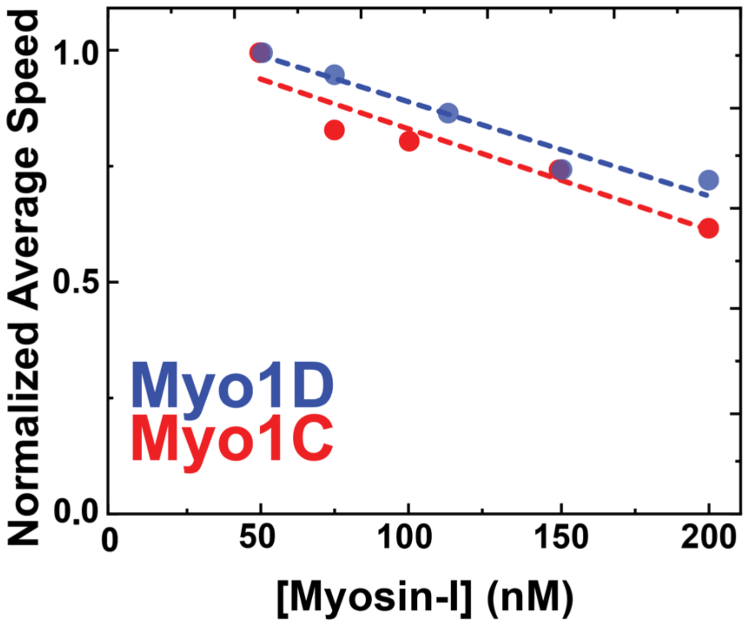

Figure S2. Normalized Average Speeds for biotinylated myosin-Is actin gliding assays. Average speeds of biotinylated myosin-Is gliding assays were normalized to observe possible sharp decrease corresponding to force sensitivity, but both myo1C (red) and myo1D (blue) have similar slopes of -0.002 ± 2.683 and -0.002 ± 4.174, respectively.

Movie S1. Video showing 10 nM myo1C and 5μM SUV (magenta; left) and 10 nM myo1D and 5 μM SUV (magenta; right) in the presence of immobilized actin filaments (green). Scale bar: 2 μm.

Reference

1. Speder, P., and Noselli, S. (2007) Left-right asymmetry: class I myosins show the direction Curr Opin Cell Biol **19**, 82-87 10.1016/j.ceb.2006.12.006
